# Accuracy of D-Dimers to Rule Out Venous Thromboembolism Events across Age Categories

**DOI:** 10.1155/2010/185453

**Published:** 2010-07-27

**Authors:** G. Der Sahakian, Y. E. Claessens, J. C. Allo, J. Kansao, G. Kierzek, J. L. Pourriat

**Affiliations:** Emergency Department, Hôtel Dieu-Cochin Hospital, Paris Descartes University, Assistance Publique-Hôpitaux de Paris, Place du Parvis Notre-Dame, 75004 Paris, France

## Abstract

*Background*. Strategies combining pretest clinical assessment and D-dimers measurement efficiently and safely rule out venous thromboembolism events (VTE) in low- and intermediate-risk patients. *Objectives*. As process of ageing is associated with altered concentrations of coagulation markers including an increase in D-dimers levels, we investigated whether D-dimers could reliably rule out VTE across age categories. *Method*. We prospectively assessed the test performance in 1,004 patients visiting the emergency department during the 6-month period with low or intermediate risk of VTE who also received additional diagnostic procedures. *Results*. 67 patients had VTE with D-dimers levels above the threshold, and 3 patients displayed D-dimers levels below the threshold. We observed that specificity of D-dimers test decreased in an age-dependent manner. However, sensitivity and negative predictive value remained at very high level in each age category including older patients. *Conclusion*. We conclude that, even though D-dimers level could provide numerous false positive results in elderly patients, its high sensitivity could reliably help physicians to exclude the diagnosis of VTE in every low- and intermediate-risk patient.

## 1. Introduction

Venous thromboembolism events (VTE) are common disorders with major impact on clinical and economic outcomes [1]. As a result, diagnostic procedures have been developed to safely rule out the occurrence of VTE especially in the setting of emergency medicine. Several studies have underlined that D-dimers level below 500 ng/ml reliably excluded the diagnosis of VTE in patients previously identified by clinical pretest evaluation as low- or intermediate-risk patients [2–7]. The use of combined strategies including both clinical assessment and D-dimers measurement have been endorsed by most professional societies as they improve diagnosis with acceptable cost efficiency. As frequency of VTE increases with age, diagnostic strategies should be adjusted to elderly age groups of patients. However, interpretation of D-dimers level is altered in older patients, as ageing or underlying clinical conditions may activate coagulation. Consequently, performance of diagnostic strategies using D-dimers varies with age. In guidelines released in 2000, the European Society of Cardiology recommended the avoidance of D-dimers measurement in patients older than 80 years of age [8]. We therefore assessed the clinical value of D-dimers test across age categories in patients presenting in the emergency department with low- or intermediate-risk of VTE.

## 2. Materials and Methods

We conducted a prospective, observational, and single centred study in the emergency department of a tertiary-level teaching hospital during the 6-month period. Study protocol and procedures complied with the principles of the Declaration of Helsinki. The institutional review board for the protection of human subjects in our hospital approved the study protocol and patients information procedures (study notification letter).

We enrolled all consecutive consenting adults (18 years old or above) who required D-dimers blood test for the diagnostic purposes of venous thromboembolism events (VTE), that is, deep venous thrombosis (DVT) and pulmonary embolism (PE). Diagnostic probability of VTE was based on the Wells score dedicated to DVT or PE, as required [3–6]. 

Patients were excluded if they refused to participate in the study, if they were less than 18 years of age, if they were pregnant, if they were perceived as a high risk for VTE after calculation of the Wells score, or if they were lost to follow-up at the end of the study period. 

Patients' management was based on currently recommended practice guidelines. Briefly, physicians were educated on diagnostic procedures for DVT and PE including the Wells pretest clinical scoring system, and computerized procedures were available at the bedside. According to Wells scores, D-dimers blood levels were required for the patients indicating low or intermediate probability for VTE. The attending physician made the score-based decision and proceeded to baseline data collection through patient interviews and standardized review of medical records. Baseline data consisted of demographic data (age, gender), coexisting illnesses, symptoms, and Wells score calculation for VTE probability. Patients were stratified by their age in the following eight age cohorts: <30 years of age (years), 31–39, 40–49, 50–59, 60–69, 70–79, 80–89, and >90 years. Pulmonary angiography on a multislice computerised tomography (CT) scan and lower limb compression ultrasonography were guided by procedures. Patients were classified as VTE or non-VTE after the diagnostic procedures, if required, and at the end of a 3-month followup.

D-dimers were measured using the ELISA quick test VIDAS. Patients were positive for D-dimers if the level was >500 ng/ml; otherwise, patients were considered negative for D-dimers.

Our objective was to assess the overall effectiveness of D-dimers levels to detect VTE across age categories in patients with low and intermediate risk of VTE. A value of *P* < .05 was considered statistically significant. Comparisons between patients with or without VTE were made by the *χ* ^2^ test for categorical variables and the Mann Whitney test for continuous variables. All variables were analyzed based on their association with the presence of a thrombosis (>500 ng/ml) having or not a VTE, using Fisher's exact test. Sensitivity, specificity, negative predictive value of D-dimers, and their 95% confidence interval (95%CI) were calculated for each age class.

All tests were two-sided, and *P*-values below  .05 were considered statistically significant. All statistical analyses were performed using SAS software V9.1 (SAS institute, Cary, NC).

## 3. Results

During the study period, 1,042 patients were enrolled; 38 could not be evaluated for the primary end point and 1,004 could be evaluated at 3 months for both D-dimers level and the presence of PE or DVT. Among these, 539 were negative for D-dimers and 465 were positive. Mean age was 74 years (19–99 years) for patients positive for D-dimers, and 48 years (18–93 years) for patients negative for D-dimers (*P* <  , 001). Only 12% of patients aged 70 or above had negative D-dimers. In the group of patients positive for D-dimers, 67 (14%) had a diagnosis of either DVT (*n* = 15, 3%) or PE (*n* = 52, 11%). In patients with negative D-dimers, 3 were identified with VTE at the end of the followup (one patient with DVT, two with PE). We observed that incidence of VTE roughly increased with age, corresponding to 5% or less in the younger patients, and reaching 13% in 80 to 89 age cohort. 

Overall sensitivity and specificity of D-dimers were 95,7% [95% CI: 0,880–0,991] and 57,4% [95% CI: 0.542–0.605], respectively. Negative and positive predictive values were 0,994 [95% CI: 0,984–0,999] and 0,144 [95% CI: 0.114–0.179]. The performance of D-dimers across age categories underscored that specificity was elevated in younger patients, and dramatically decreased in elderly especially in those who were 80 years or older. Conversely, sensitivity of the test remained above 80%, and the negative predictive value of D-dimers was maintained across age cohorts (Table 1).

## 4. Discussion

Coagulation is a tightly amplified and regulated process. It generates an intricately related network that contributes to the adequate response against the primary insult [9]. During the ageing, these mechanisms are deregulated. More precisely, with ageing, there is an imbalance in the coagulation cascade that navigates towards a prothrombotic environment. This is supported by our study that reported an increasing number of VTE with ageing, with a predominance of PE. 

Several studies reported pronounced activation of coagulation system in older patients characterized by higher levels of fibrinogen, factors VII, VIII, IX, and other clotting factors [10, 11]. The analysis of a large cohort from the North American Cardiovascular Health Study pointed out the association between clotting and inflammatory markers and frailty in elderly population even in the absence of clinically relevant comorbidity [12]. In 1995, Mari et al. reported that baseline coagulation activity physiologically increased in centenarians with increased thrombin concentration and elevated level of D-dimers [10]. These data predicted the weak significance of D-dimers elevation in older patients.

Few elderly patients were included in the studies reporting procedures to rule out VTE using a decision making process based upon D-dimers threshold. In 2001, Wells et al. published the strategy for diagnosis of PE in patients whose mean age was 50.5 years (18.4), ranging from 16 to 93 years [4]. Two years later, the same authors published an approaching strategy in patients with suspected DVT whose mean age was 58 years [3]. These cornerstone studies did not specifically assess the relevance of these strategies in older patients. Rathbun et al., in 2004, evaluated such procedures in patients that were from 19 to 83 years [7]. One prospective and three retrospective studies previously assessed the performance of D-dimers in elderly patients visiting the emergency department with suspected PE [13–17]. These studies used various methods for D-dimers measurement. Sensitivity was almost 100% [14, 15] across all age groups, and the negative predictive value remained clinically relevant in elderly, ranging from 82.4% to 100%. However, specificity decreased below 50% after 70 years, dropping to 14.3% in the very old patients. This suggested that D-dimers should be used cautiously to rule out, diagnosis of VTE in elderly population. Additionally, increasing D-dimers cutoff resulted in an unacceptable loss of sensitivity with a marginal increase of specificity [11, 13, 17]. 

In this paper, we have demonstrated that negative predictive value of D-dimers measurement using the reference method efficiently ruled out the diagnosis of VTE in low- or intermediate-risk patients across age categories whereas specificity decreased in an age-dependent manner. The overall sensitivity of D-dimers was relatively low—95,7% [95%CI 0.880–0.991]—as compared to previous studies whose sensitivity was nearly 100%. Conversely, we observed a high overall specificity of D-dimers that dramatically dropped in the older patients, as previously reported. A former study reported decreased sensitivity (22% to 14%) and specificity (31% to 22%) in patients older than 75 years [16]. Similar results were observed in patients older than 70 years [13, 14]. In our paper, the number of patients with D-dimers level above the threshold increased with age, and only 3% of patients above 90 years had negative D-dimers.

Since specificity of D-dimers was poor in patients older than 80, the European Society of Cardiology [8] recommended that D-dimers are not to be used in the latter population no matter what pretest risk category for VTE is. However, negative D-dimers still rule out VTE in older patients. As PE occurs most frequently and since ultrasounds are of a minor help in this condition, our results as well as other results point out the applicability of the rule-out strategies for PE in the frailest elderly population. Rule-out strategies could spare the elderly patients from potentially detrimental investigations requiring intravenous radiocontrast [18].

This study has several limitations. We conducted a single centre study; patients were excluded if they were perceived as a high risk for VTE after calculation of the Wells score; the number of consecutive patients enrolled in this study was not evaluated *a priori*; in addition, the number of patients is heterogeneous across the age cohorts and some age cohorts, are probably underrepresented.

## 5. Conclusion

In conclusion, we recommend the use of D-dimer testing in elderly patients to rule out pulmonary embolism in low- and intermediate-risk patients where its negative predictive value and sensitivity remain 100%.

## Figures and Tables

**Table 1:** Performance of the D-dimers “Vidas” assay according to age categories in 1,004 patients with suspected venous thromboembolic events (VTE). 95%CI: 95% confidence interval.

Age categories (years)	No. of patients	No. of VTE	Sensitivity [95%CI]	Specificity [95%CI]	Negative predictive value [95%CI]
<30	91	5	80% [28.4–99.5]	83.5% [74.3–90.5]	98.7% [93.0–100]
30–39	121	5	80% [28.4–99.5]	75.6% [67.0–83.0]	98.9% [94.0–100]
40–49	151	7	100% [59.0–100]	78.7% [71.3–84.9]	100% [96.9–100]
50–59	155	6	83.3% [35.9–99.6]	69.2% [61.4–76.4]	99.1% [95.0–100]
60–69	142	11	100% [71.5–100]	56% [47.1–64.5]	100% [95.2–100]
70–79	165	14	100% [76.8–100]	33.3% [26.1–41.1]	100% [93.4–100]
80–89	134	18	100% [81.5–100]	10.1% [5.3–17.0]	100% [73.5–100]
>90	45	4	100% [39.8–100]	7.3% [4.0–20.0]	100% [29.2–100]

All patients	1,004	70	95.8% [88.0–99.0]	57.4% [54.2–60.5]	99.4% [98.4–99.9]

## References

[1] White RH (2003). The epidemiology of venous thromboembolism. *Circulation*.

[2] Wells PS, Anderson DR, Rodger M (2003). Evaluation of D-dimer in the diagnosis of suspected deep-vein thrombosis. *The New England Journal of Medicine*.

[3] Wells PS, Anderson DR, Rodger M (2001). Excluding pulmonary embolism at the bedside without diagnostic imaging: management of patients with suspected pulmonary embolism presenting to the emergency department by using a simple clinical model and D-dimer. *Annals of Internal Medicine*.

[4] Wells PS, Owen C, Doucette S, Fergusson D, Tran H (2006). Does this patient havedeepvein thrombosis?. *Journal of the American Medical Association*.

[5] Manfredini R (2006). D-dimer for the diagnosis of acute venous thromboembolism in the emergency department: a Janus-face marker. *Internal and Emergency Medicine*.

[6] Imberti D (2007). D-dimer testing: advantages and limitations in emergency medicine for managing acute venous thromboembolism. *Internal and Emergency Medicine*.

[7] Rathbun SW, Whitsett TL, Vesely SK, Raskob GE (2004). Clinical utility of d-dimer in patients with suspected pulmonary embolism and nondiagnostic lung scans or negative CT findings. *Chest*.

[8] Büller HR, Agnelli G, Hull RD, Hyers TM, Prins MH, Raskob GE (2004). Antithrombotic therapy for venous thromboembolic disease: the Seventh ACCP Conference on Antithrombotic and Thrombolytic Therapy: evidence-based guidelines. *Chest*.

[9] Opal SM, Esmon CT (2003). Functional relationships between coagulation and the innate immune response and their respective roles in the pathogenesis of sepsis. *Critical Care*.

[10] Mari D, Mannucci PM, Coppola R, Bottasso B, Bauer KA, Rosenberg RD (1995). Hypercoagulability in centenarians:the paradox of successful aging. *Blood*.

[11] Sagripanti A, Carpi A (1998). Natural anticoagulants, aging, and thromboembolism. *Experimental Gerontology*.

[12] Cohen HJ, Harris T, Pieper CF (2003). Coagulation and activation of inflammatory pathways in the development of functional decline and mortality in the elderly. *American Journal of Medicine*.

[13] Tardy B, Tardy-Poncet B, Viallon A (1998). Evaluation of D-dimer ELISA test in elderly patients with suspected pulmonary embolism. *Thrombosis and Haemostasis*.

[14] Righini M, Goehring C, Bounameaux H, Perrier A (2000). Effects of age on the performance of common diagnostic tests for pulmonary embolism. *American Journal of Medicine*.

[15] Righini M, de Moerloose P, Reber G, Perrier A, Bounameaux H (2001). Should the D-dimer cut-off value be increased in elderly patients suspected of pulmonary embolism ?. *Thrombosis and Haemostasis*.

[16] Söhne M, Kamphuisen PW, Van Mierlo PJWB, Büller HR (2005). Diagnostic strategy using a modified clinical decision rule and D-dimer test to rule out pulmonary embolism in elderly in- and outpatients. *Thrombosis and Haemostasis*.

[17] Harper PL, Theakston E, Ahmed J, Ockelford P (2007). D-dimer concentration increases with age reducing the clinical value of the D-dimer assay in the elderly. *Internal Medicine Journal*.

[18] Levin ML (1995). Acute renal failure in the elderly: strategies for prevention. How the physiologic effects of aging increase nephrotoxic risk. *The Journal of Critical Illness*.

